# A novel perceptual trait: gaze predilection for faces during visual exploration

**DOI:** 10.1038/s41598-019-47110-x

**Published:** 2019-07-24

**Authors:** Nitzan Guy, Hagar Azulay, Rasha Kardosh, Yarden Weiss, Ran R. Hassin, Salomon Israel, Yoni Pertzov

**Affiliations:** The Hebrew University of Jerusalem, Jerusalem, Israel

**Keywords:** Social neuroscience, Social behaviour, Perception, Human behaviour

## Abstract

Humans are social animals and typically tend to seek social interactions. In our daily life we constantly move our gaze to collect visual information which often includes social information, such as others’ emotions and intentions. Recent studies began to explore how individuals vary in their gaze behavior. However, these studies focused on basic features of eye movements (such as the length of movements) and did not examine the observer predilection for specific social features such as faces. We preformed two test-retest experiments examining the amount of time individuals fixate directly on faces embedded in images of naturally occurring scenes. We report on stable and robust individual differences in visual predilection for faces across time and tasks. Individuals’ preference to fixate on faces could not be explained by a preference for fixating on low-level salient regions (e.g. color, intensity, orientation) nor by individual differences in the Big-Five personality traits. We conclude that during visual exploration individuals vary in the amount of time they direct their gaze towards faces. This tendency is a trait that not only reflects individuals’ preferences but also influences the amount of information gathered by each observer, therefore influencing the basis for later cognitive processing and decisions.

## Introduction

When we explore complex scenes, we frequently move our gaze to different locations within our visual surroundings. By moving our gaze, we select which part of the environment will be processed by the center of the retina (i.e. the fovea) that consists of a dense distribution of photoreceptors. During gaze exploration we often return to already sampled objects, such resampling leads to accumulation of information and therefore influences high-level cognitive and social processes (e.g., visual memory^1^ and emotion recognition^2^). In the last century, a growing number of eye tracking studies have examined the factors involved in determining gaze position. Early studies identified three main factors: the task, the stimuli, and the observer^3,4^. For example, Yarbus (1967), in his seminal studies showed that when observers perform different tasks (e.g. assessing peoples’ age vs remembering the position of the people in the room), their scanning patterns are completely different^4^. In addition, as an example to the stimuli factor, he has shown that when observing faces, gaze is directed mostly to a triangle restricted by the eyes and mouth, and larger eyes attract more fixations. Regarding to the observer factor Yarbus noted that “it may be concluded that individual observers differ in the way they think and, therefore differ also to some extent in the way they look at things”^4^. To date, the vast majority of eye tracking studies have focused on task and stimuli-specific effects but have largely ignored the observer factor: how different individuals move their gaze differently even when confronted with the same task and stimuli. This variability was often treated as noise and data was collapsed across observers. However, as different individuals exhibit distinct and reliable characteristics of gait and speech^5,6^, one would expect the existence of distinct characteristics in gaze behavior. Unlike gait and speech, gaze behavior strongly determines the visual input arriving in the brain, making such variability an important factor in determining and reflecting one’s inner world. In the current study we report on a novel gaze related trait that influences the amount of social information accumulated by the observer. More specifically, we show that the amount of time subjects fixate on others’ faces (face-preference) varies between individuals in a reliable manner.

A handful of recent studies examined the variability of eye movements between observers using test-retest procedures^7–9^. This procedure enabled researchers to distinguish which characteristics are stable and reliable, and therefore should be treated as a trait of the observer rather than “noise”. Unlike our interest in face-preference, almost all of the studies that aimed to identify meaningful individual differences in eye movement patterns have focused on basic eye movement characteristics (during scene exploration^7^ and reading^7,10^). For example, in one study^7^ participants performed four tasks: scene memorization, text reading, visual search and pseudo-text reading^7^. Each participant performed each task twice, several days apart. Mean saccade amplitude (i.e., the distance of the rapid and frequent gaze movements, termed saccades) and mean fixation duration (i.e., the duration in which gaze is relatively stable between saccades) differ between individuals. Moreover, these measures were consistent across different tasks and across several days. These studies highlight an interesting and consistent pattern of variation, systematic individual differences in generalized features underlying gaze behavior, suggesting that individuals vary in their capacity to scan their environment for visual information.

While these studies addressed differences in scanning behavior that do not relate to the observed stimuli, less is known regarding individual differences in gaze behavior with respect to the observed content. For example, do different observers exhibit different predilection to fixate on specific type of stimuli? That is, regardless of the differences in exploration metrics, such as saccade amplitude, do people differ systematically in the content they fixate upon? If such a reliable difference exists, it means that different participants incorporate some kind of “perceptual trait” that influences the amount of information they accumulate on specific contents in their environment. Such traits may lead to entirely different experiences and memories^1^ from a given situation, and these are expected to affect higher level processes such as planning and decisions.

The only study that addressed individual differences in content-based gaze behavior was performed on a specific type of stimuli and task^8^. In their study, Mehoudar and colleagues (2014) asked participants to memorize and then recognize static images of isolated faces. They showed that some observers fixate mainly on lower face regions such as the mouth and nose and some fixate mainly on the eyes. These differences between individuals were found to be consistent across 18 months.

Although most of these previous studies did not explore the relationship between individual differences in gaze behavior and image content, it is well established that the content of images strongly affects how people explore them^3,4^. Moreover, previous studies have shown a common tendency to fixate on socially relevant information, such as faces. For example, social features, especially faces, are more strongly preferred by the observers compared to other highly salient regions^11^, a phenomenon which appears to be independent of the observers’ task^12^. This preference begins early in life, and has even been observed in infants as young as 6 months old^13^. These findings are consistent with vast evidence regarding the premium placed on faces as a key element involved in social processes, such as in social interaction and understanding of others’ emotions^14,15^. Importantly, many factors of the faces influence the observers’ scanning patterns. For example: direction of gaze^16,17^, emotional expression^18,19^, facial attractiveness^20,21^ and configurational aspects of faces^22,23^. However, these studies focused on the commonalities across observers and did not examine how observers differ in their scanning patterns. In the current study, we were interested in exploring the general tendency of each observer to fixate on faces (a term we denote here as face-preference), regardless of their exact facial features. Therefore, we included variety of images that differ in the number of faces in them and in their facial cues. The use of a variety of pictures with variable types of faces, expressions and configurations is part of an endeavor to explore gaze behavior in more ecological scenarios^24^.

Not only socially relevant information attracts gaze, it has been shown that visually salient areas also attract our gaze. Early models tried to predict the probability of fixating on different locations using low level visual features (such as orientation, color and intensity). The map that combines spatial contrast of various low-level features is often termed “saliency map”^25,26^. De facto, the low level explanation of gaze position relies on bottom-up processes without referring to the semantic content of the images. More recent models included the impact of higher level features such as text and faces^27–31^. The inclusion of these high level factors represents the additional contribution of top-down processes to visual exploration of scenes. Fecteau and Munoz^27^ (2006) suggested that both saliency (low-level features) and content are critical for adequate explanation of selected gaze position. Moreover, they showed that not only the content of the stimuli is important, but the features that are relevant to the observer’s task influence gaze more than unrelated features. Maps that combine both saliency, high level semantics and goals are often termed “priority maps”^32^. Importantly, all these models take into account the impact of the visual input and task but neglect the observer factor: observers differ in their scanning pattern, even when confronted with identical stimuli and task. Thus, to improve the existing models, models should take into account the unique preference of the observer, better reflecting his\her unique gaze behavior. If face-preference is a reliable trait of an observer, it should be considered in future gaze behavior models. In order to account for the possibility that face-preference is a result of the preference to fixate on regions that are conspicuous in their visual saliency (low level features), we explored the individual differences in saliency-preference and examined whether it could predict face-preference.

In experiment 1 we examined whether individual differences in face-preference and saliency-preference are stable across time. Observers were presented with identical images in two sessions taken an hour apart. When identical images are observed, similar scanning patterns are expected to occur^33,34^, therefore stable individual differences in face-preference and saliency-preference might be explained by stimulus specific factors (the specific scanning patterns that occur on specific images, or the specific facial features in this set of pictures). In addition, by presenting the same images twice some aspects of gaze behavior might change due to familiarity or stimulus specific habituation^35,36^. Therefore, in order to assess whether this variability is observed across different stimuli and different task demands we conducted experiment 2. In experiment 2, participants were instructed to memorize arousing images which differed across two sessions. Importantly, in both experiments we used a task that allows participants to move their eyes freely. Finding reliable individual differences in face and saliency preferences in both experiments would strongly suggest that there are indeed stable individual differences in gaze preferences that do not relate to the specific images that were observed.

Finally, in an attempt to delineate key factors that relate to face-preference, we tested the degree to which individual differences in face-preference are correlated with personality traits. There is a broad consensus among personality researchers that individual differences in characteristic patterns of feelings, thoughts, and behavior – otherwise termed personality - can be categorized along five broad dimensions^37–40^. These dimensions, called the ‘Big Five’, are: Agreeableness, Conscientiousness, Extraversion, Neuroticism and Openness-to-experience. As measured by self-report questionnaires designed to assess the Big-Five dimensions, individual differences in personality traits are correlated with a panoply of behavioral measures, such as face recognition^41^ and processing emotional stimuli^42^. Therefore, in this study we explored whether the variability between participants in their face-preference is related to individual differences in the Big Five. If face preference is related to personality traits, we expect to find a correlation between the two in all experimental sessions. If this relationship is not robust, or evident only in specific context, it should not be observed in all sessions. Moreover, in Experiment 1, in order to capture more specific sociability-related individual differences which may relate to gaze preferences for faces, participants also filled a number of additional questioners known to be related to interpersonal functioning. These included, measures of social values (Social Value Orientation^43^, Social Dominance Orientation^44^), empathy (Interpersonal Reactivity Index^45^), depression, social anxiety (as assessed by the Beck Depression Inventory^46^, and Social Phobia Inventory^47^), and autism spectrum disorder symptomatology (as assessed by the Autism Quotient^48^).

In summary, the goal of this study was to test whether there are stable individual differences in gaze preference to faces and saliency (face-preference and saliency-preference) across time and whether these differences can be predicted by other individuals’ traits.

## Experiment 1

In Experiment 1, each participant freely viewed images of complex scenes in two sessions taken an hour apart. Each session consisted of the same 40 scenes that contain faces. Each image was displayed for 3 seconds. Participants filled the Big-Five questionnaire a few days before the experiment^49^.

### Results

Individual differences in gaze behavior is still a relatively unexplored field. Therefore, in addition to our main hypothesis, we were interested in replicating previous findings showing that basic gaze behavior characteristics (fixation duration and saccade amplitude) are stable across time^7^. As expected, we found that both fixation duration and saccade amplitude are reliable across sessions (see Supplementary Materials).

#### Internal consistency – correlations within a session

Internal consistency of individual differences in face-preference and saliency-preference was assessed by comparing each preference value (faces and saliency) in odd vs. even trials. Preference values were defined as the percentage of time in which gaze was directed to parts of the image with above threshold values in each feature, saliency or faces (see methods). In other words, participants with higher face-preference are those who fixated more on faces, while participants with higher saliency-preference might be fixating more on bold colors or prominent contours. Importantly, although the same images appeared in both sessions, within each session each image appeared only once. Therefore, by comparing even and odd trials we compared two different sets of images. Pearson correlations of face-preference scores were significantly positive in both sessions (session 1: r(28) = 0.66, p < 0.001; session 2: r(28) = 0.57, p = 0.001). However, saliency-preference scores were not consistent across odd and even trials (session 1: r(28) = −0.19, p = 0.319; session 2: r(28) = −0.19, p = 0.339). To further investigate the internal consistency across other devisions of the data set, we performed a permutation analysis. We divided the stimuli 1000 times into two equal data sets of images and examined the correlation between face-preferences in each of those two sets. All 1000 correlations were significantly above zero (mean-r = 0.88, std-r = 0.04; see Supplementary Fig. S6). Addionally, we performed the same analysis for saliecny-preference. Around eight percent of the correlaions were significantly larger than 0 (mean-r = 0.15, std-r = 0.15; see Supplementary Fig. S7). High internal consistency is necessary in order to argue that a preference is a trait of the observer. However, it is not sufficient, as strong internal consistency can also be explained by the current state of the observer. Therefore, we also examined reliability of preference values across different testing sessions.

#### Test-retest reliability – correlations between sessions

**Figure 1:**
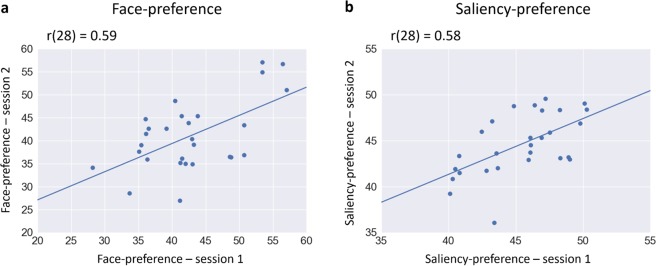
Scatter plots and regression lines between individuals’ preference values in the first and second sessions. The values represent the percent of fixation time on faces (**a**) and low-level salient regions (**b**).

The temporal stability of individual differences in preference values was assessed by comparing each preference value (faces and saliency) in the first session and the second session (see Supplementary Table S1). Results of the Pearson correlations indicated a significant positive association in face-preference (r(28) = 0.59, p = 0.001) and saliency-preference (r(28) = 0.58, p = 0.001) between sessions (Fig. 1). These findings suggest that individuals vary in their preferences in a reliable manner.

Note that in this comparison both data sets consist of identical images, therefore high correlations can be a result of similar gaze behavior when viewing the same images twice. For example, a specific participant might find one face, or facial feature, in a specific image more attractive and look at it longer. Because the same image was presented also in the second session, this participant would exhibit relatively high face-preference that is related to the specific image or facial feature. To further validate the reliability of face-preference variation in different sets of images, we performed permutation analysis. We divided the whole set of images 1000 times into two different data sets with equal number of images leading to 1000 pairs of data sets with different images. Then we examined correlation between individuals’ face-preferences in each pair of sets. Importantly, one face-prefernce value reflected gaze behavior in session 1 and the other in session 2. Ninety three percent of the correlations were significantly larger than 0 (mean-r = 0.49, std-r = 0.08; see Supplementary Fig. S6). Thus, strong correlations in face-preference were observed between individuals’ gaze preference in two different sets of images, regardless of the exact images within each set. To complete the picture, we tested saliency-preference as well. Twenty two percent of the correlations were significantly larger than 0 (mean-r = 0.26, std-r = 0.15; see Supplementary Fig. S7). This suggests that the reliability in saliency preference is somewhat dependent on the exact set of pictures that observers see.

Next, for each preference value we performed paired sample t-tests between the two sessions in order to examine if subjects’ preferences differ in a consistent manner in the two sessions. No significant differences between the two sessions were observed in both faces and saliency preferences (Faces: t(27) = 1.36, p = 0.186; Saliency: t(27) = 1.37; p = 0.18).

#### Relations between saliency-preference and face-preference

Faces are typically more visually salient than their surroundings, therefore it is possible that participants with high preference for salient regions would also fixate more on faces due to saliency preference rather than predilection for faces. To check whether the variability in face-preference could be explained by saliency-preference we performed partial correlation tests. First, we correlated face-preference in session 1 and session 2 when controlling for saliency-preference in session 1. Reliability of face-preference prevails even when controlling for the preference to low level features in session 1 (r(25) = 0.59, p = 0.005). Thus, the correlation between face-preference in session 1 and 2 could not be explained by preference to fixate on low level salient features.

Next, we checked whether the correlation of saliency-preference between sessions could be explained by face-preference. We performed partial correlations between the saliency-preference of the two sessions, this time controlling for the face-preference in session 1 (r(25) = 0.53, p = 0.004). The results support the existence of two independent preferences: one for low level saliency and the other for faces.

#### Correlation with personality measures

To assess the relation between personality traits and preferences values (faces and saliency), we examined the Pearson correlations between the average preferences values in both sessions and each personality trait (see Supplementary Table S2). Only one significant association was found between conscientiousness and face-preference (r(28) = −0.47, p = 0.01), suggesting more conscientiousness observers exhibit lower face-preference. This association is not significant when performing Bonferroni correction for multiple comparison, and therefore should be considered cautiously. In order to further examine this relation relationship between personality traits and face preference we repeated it in experiment 2.

In this experiment participants were also asked to fill in a few questionnaires in addition to the Big-Five questionnaire: Beck Depression Inventory^46^ (BDI), Social Phobia Inventory^47^ (SPIN), Social Value Orientation^43^ (SVO), Social Dominance Orientation^44^ (SDO), Autism Quotient^48^ (AQ), Interpersonal Reactivity Index^45^ (IRI). We examined the relation between face and saliency preferences to these questionnaires and did not reveal any significant relation (see Supplementary Table S3). Therefore, participants in experiment 2 did not fill these questionnaires. Finally, we checked if the age of the observers is related to the average face-preference in both sessions. Observers’ age was not significantly correlated with face-preference (r = 0.30, p = 0.117). Note, that age is highly restricted in our experiment (range: 23–29), therefore, our results only suggest that within this range - age does not influence face-preference.

### Discussion

Using a test-retest procedure we report on a novel perceptual trait: predilection for fixating on faces. This experiment unveils a substantial and reliable variability between individuals’ face-preference which determines the amount of socially relevant information that gets to individuals’ brains. Face preference may be related to the conscientiousness personality factor. Other questionnaires and personality traits do not seem to relate to face-preference.

Importantly, experiment 1 revealed that only face-preference was stable within a session (in both split-halves analysis and permutation analysis) when the two image sets were different. On the other hand, across sessions that consisted of identical images, saliency-preference was stable as well. This discrepancy may be a result of presenting the same images in both sessions, which may result in similar gaze behavior that depend on the specific displayed images. In addition, we examined whether the variability in face-preference is a result of a preference to specific collection of images and facial cues. We performed a permutation analysis in which each data set is splitted to 1000 different divisions into two data sets. If specific facial cues are the source of the variability in face-preference, we would expect some divisions to be imbalance (in terms of the specific facial cues) and to show non-significant correlations. However, we found strong and reliable internal consistency in face preference in all divisions.

In order to further validate that the reliability we report is not specific to the selected images and not dependent in the short interval between sessions, in experiment 2 we used different picture sets in each session, taken weeks apart (all over one month apart). Moreover, in experiment 2 participants performed a memory task in order to validate that the reported stability is evident in different viewing conditions.

## Experiment 2

In experiment 2, each participant completed two scene memorization tasks taken weeks apart. In each session participants viewed 80 scenes containing faces in emotional situations. Each image appeared for 3 seconds. In contrast to experiment 1, in this experiment participants viewed a different set of images in each session. Following the task, the participants filled in the Big-Five questionnaire^50^. As in experiment 1, we analyzed observers’ specific face-preference and saliency-preference and how the two are related to personality traits.

### Results

As in experiment 1, we examined whether basic gaze behavior characteristics are reliable. Consistent with previous studies^7^ and experiment 1, mean fixation duration and saccadic amplitude were found to be reliable across sessions (see Supplementary Materials).

#### Internal consistency – correlations within sessions

**Figure 2:**
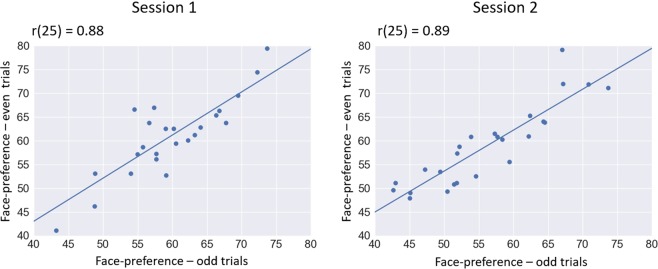
Scatter plots and regression lines between individuals’ preference values in odd and even trials in both sessions.

The internal consistency of individual differences in preference values was assessed by comparing each preference value (faces and saliency) in the odd and even trials. Significant positive correlations were observed in face-preference in both sessions, as seen in Fig. 2 (session 1: r(25) = 0.88, p < 0.001, session 2: r(25) = 0.89, p < 0.001). Saliency-preference correlation was marginally significant in session 1 (r(25) = 0.38, p = 0.062) and significant in session 2 (r(25) = 0.54, p = 0.006). Similar to experiment 1, we performed permutation analysis to examine internal consistency across various devisions of the data set. We divided the stimuli 1000 times into two data sets and examined the correlation between face-preferences in the two sets. We found all 1000 correlations to be significantly larger than zero (mean-r = 0.75, std-r = 0.06; see Supplementary Fig. S6). Addionally, we performed the same analysis for saliecny-preference. Ninety percent of the saliency internal consistency correlation values were significantly higher than zero (mean-r = 0.59, std-r = 0.15; see Supplementary Fig. S7).

#### Test-retest reliability – correlations between sessions

**Figure 3:**
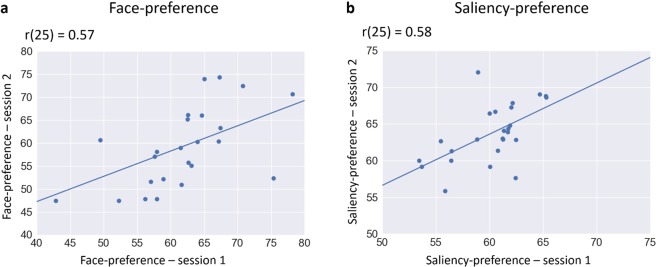
Scatter plots of face (**a**) and saliency (**b**) preference in the first versus the second sessions.

The stability of individual differences in preference values was assessed by comparing each preference value (faces and saliency) in the first and the second sessions (descriptive tables in Supplementary). Significant positive correlations were found in face-preference (r(25) = 0.57, p = 0.003) and saliency-preference (r(25) = 0.58, p = 0.002) as illustrated in Fig. 3.

These findings further support the claim that individuals vary in their saliency and face preferences in a reliable manner, even when tested weeks apart. To further investigate the reliabilty in different sets of images, we performed a permutation analysis. Similar to experiment 1, we divided the stimuli set 1000 times into two sets of images and examined the correlation between individuals’ face-preferences in each one of the two sets (Session 1 – first set; Session 2 – second set). Ninety eight percent of the 1000 correlation values were significantly larger than zero (mean-r = 0.53, std-r = 0.06; see Supplementary Fig. S6). Then, we tested saliency-preference as well. Half of the correlations were significantly larger than zero (mean-r = 0.39, std-r = 0.14; see Supplementary Fig. S7).

Next, we performed paired sample t-tests to check for consistent differences between session 1 and 2. No significant difference was observed in face-preference (Faces: t(24) = 1.35, p = 0.189). Saliency-preference was significantly different in the two sessions (t(24) = -5.28; p < 0.001), suggesting that observers are more attracted to low level saliency in the second session. Note that these differences might be a result of the different set of images used in session 2. Importantly, face-preference was stable between sessions although the images were emotional and different in the two sessions.

In experiment 2 the time between sessions varied between participants, therefore we examined whether the length of the interval between sessions influences the consistency in face preference. The difference in face-preference between the two sessions was not correlated with the length of the interval between sessions (r = 0.11, p = 0.6). Thus, the stability in face-preference does not seem to be affected by the duration of time that passes between sessions.

#### Relations between saliency-preference and face-preference

To check whether the variability in face-preference could be explained by preference to low level saliency we performed partial correlation tests. Similar to experiment 1, we correlated individuals’ face-preference in session 1 and session 2, controlling for saliency-preference in session 1. Reliability of face reference prevails even when controlling for the saliency-preference (r(20) = 0.52, p = 0.009). The results suggest that the correlation between face-preference in session 1 and session 2 could not be explained by saliency-preference.

Next, we investigated whether the correlations in saliency-preference could be explained by face-preference. We performed a partial correlation between the saliency-preference in both sessions, controlling for face-preference in session 1 (r(22) = 0.26, p = 0.216). The results suggest that, in this experiment, individual differences in saliency preference could be explained by face-preference.

These findings validate the findings in Experiment 1 that implicated face-preference as a reliable trait that could not be explained by saliency preference.

#### Correlation with personality measures

To check the relation between personality traits and preferences values (faces and saliency), we performed Pearson correlations between the average preference values in both sessions and each personality trait. Full correlation matrix with all the Big-Five domains is depicted in the Supplementary Materials. The correlation between conscientiousness and face-preference was not significant (r(25) = 0.16, p = 0.436) and in the opposite direction to the one found in experiment 1.

In addition, we examined whether greater face-preference or saliency-preference are related to memory performance, as assessed by a final recognition block with 10 old and 10 new images. Correlations between memory performance and face-preference (r(25) = −0.22, p = 0.282) and saliency-preference (r(25) = -0.06, p = 0.794) were not significant.

Finally, we examined if the age and sex of the observers is related to their face-preference (averaged across both sessions). No significant difference was observed between females (M = 58.9, SD = 8.6) and males (M = 61.4, SD = 6.03; t(23) = 0.77, p = 0.449). The correlation (Spearman) between age and face-preference was not significantly different than zero (r(25) = 0.01, p = 0.643).

### Discussion

The results of experiment 1 were replicated, demonstrating the existence of individuals’ distinct preference to fixate on faces, even under a memorization task of stimuli with emotional impact. In this case, test-retest reliability was measured weeks apart and on different images, providing additional support for the robustness of individuals’ preference characteristics.

As in experiment 1, face-preference was reliable even when controlling for individuals’ preference to fixate on visually salient regions (saliency-preference). Saliency-preference was also reliable across sessions, however, it could be explained by observers’ face-preference. Both saliency and face preferences internal consistency were higher in experiment 2 relative to experiment 1, which might be a result of presenting more images in each session – increasing the signal vs noise ratio. Overall both experiments indicate a stable and reliable preference for both saliency and faces.

In this experiment, personality traits were not related to face and saliency preferences. Yet, it is possible that the relation between conscientiousness and face-preference is related to the specific context and task preformed in experiment 1, and therefore absent in this task. In any matter, no general relationship between personality traits and face-preference could be observed.

## General Discussion

In two experiments individuals’ preference to fixate on faces was found to be stable across different sessions taken an hour (experiment 1) and weeks (experiment 2) apart. Thus, individuals that fixate more on faces in one set of images, tend to fixate more on faces also in other sets of images. These results demonstrate that not only basic characteristics of gaze behavior are stable^7^, but also content-based characteristics, such as face-preference, could be regarded as stable traits of human behavior. In addition, we observed a high stability in individuals’ tendency to fixate on image parts that are conspicuous in their low-level visual features (i.e. saliency-preference). Importantly, observer’s face-preference could not be explained by his\her saliency-preference, nor by any of the personality traits as measured by the Big-Five questionnaires.

Recent studies referred to the importance of image content for adequate prediction of gaze behavior, when collapsing the gaze data across participants^11,28,30^. Henderson and Hayes (2017) claimed that the selection of gaze position is better explained by a “meaning map” (a map describing which areas are meaningful, as reported by independent cohort of participants) rather than the low level saliency map of the image^30^. Our study puts forward the existence of individual differences in what is meaningful, or more precisely, in the visual predilection for faces. These individual differences between observers have two main implications. The first is an intuitive one, different visual predilection results in different visual input that influences additional visual and cognitive processes. Although we did not explore these influences in this study, we expect that face-preference should explain individual differences in other cognitive and social abilities. Second, and maybe less intuitive, is that these stable individual differences indicate that each observer “holds” a set of visual predilections that impact and control gaze behavior in a certain context. In this study we found individual differences in visual predilection for faces which constitute an important example for such individual predilection towards social information.

We tested one potential factor that may explain this variability, individuals’ personality traits, by examining the correlation between each dimension of the Big-Five model to face-preference and saliency-preference. We did find one significant association between conscientiousness and face-preference in experiment 1, suggesting more conscientiousness observer look less at faces during a free viewing task. However, a similar relationship was not observed in experiment 2. The existence of this correlation in experiment 1, but not experiment 2, could be explained by its sensitivity to the experimental design (e.g. viewing task, stimuli). Alternatively, the significant correlation may be a false positive result. Future studies should shed light whether face-preference is related to conscientiousness in some contexts. All together, we did not observe a robust link between the big-Five personality traits and face-preference which is independent of the context. Note that in experiment 1 we tested the results of several other questioners (BDI, SPIN, SDO, SVO, AQ and IRI), none explained face preference, and therefore these questioners were not repeated in experiment 2. We argue that these results do not imply that other traits, that were not examined here, are not associated with face-preference and saliency-preference.

Importantly, in this study we did not examine the different characteristics of the faces presented in the images. It might be the case that observers differ in the weights they give to different facial cues and these weights lead to different face-preference values. For example, one observer might “prefer” to look at faces with black hair and another one might prefer blond hair. If the faces in our data set are not balanced correctly (e.g. all of faces with black hair) then the variability in face preference could be explained by variability in preference towards certain facial features (e.g. hair color). However, such explanation is unlikely as high reliability was found when correlating face-preference between various sets of images. Thus, considering our permutation analysis that showed high correlation in all different divisions of the stimuli into two subsets, it is likely that there is a distinct individual tendency to look at faces, regardless of their specific facial cues.

During the review process we found out about a relevant pre-print published in bio-archives^50^. This study examined gaze behavior during initial stages of scene viewing and its relation to face recognition. They found reliable difference in face-preference as well as in preference to fixate on other semantic categories. Moreover, they measured the reliability of the proportion of first fixations on face regions and found this measure to predict face recognition performance in another task. To correspond with this article, we also performed similar analysis and tested the stability of the time it takes observers to fixate directly on a face. In our data sets this measure was found to be stable (experiment 1: r(28) = 0.36, p = 0.07; experiment 2: r(25) = 0.38, p = 0.04) but not as strong as face-preference – the amount of time fixating on faces.

This study demonstrates that face-preference and saliency-preference are unique traits of the observer. Previous studies have used these preferences to differentiate between clinical groups and controls. For example individuals with callous-unemotional traits (i.e. lack of empathy and disregarding others’ distress) or Autism Spectrum Disorders (ASD), disorders characterized by deficits in social interactions, tend to exhibit lower face-preference^51–55^. To the best of our knowledge, only Wang and colleagues (2015) examined preference for both faces and saliency^51^. In their study, individuals with ASD exhibited general increase in saliency-preference and reduced face-preference. In addition, individuals with attention-deficit hyperactivity disorder (ADHD), showed stronger saliency preference compared to controls^56^, without examining face-preference. In the current study, we focus on a typically developed student sample, nevertheless we show a considerable range of face and saliency preferences (e.g. in experiment 1, the ranges of saliency-preference and face-preference were 36–50% and 27–57% respectively). In Experiment 1, we examined autism-like-trait (assessed by the Autism Quotient^48^) in normal population and found no relation with either face-preference nor saliency-preference. In addition, we did not find a consistent pattern of association with Big-Five personality dimensions. However, future studies may benefit from examining individual differences in other social-cognitive skills within the normal range, for example measures of executive control, emotion recognition or face-identity recognition.

Another potential factor which may give rise to individual differences in face preferences is genetics. Twin studies report that visual exploration of scenes are highly heritable^57,58^. For example, Constantino and colleagues (2017) presented toddlers (18–24 months) faces and social scenes. Although they did not measure face-preference they measured both eyes-preference and mouth-preference (fixation time on eyes and mouth respectively). Eyes and mouth preferences were found to be very similar between monozygotic twins (intraclass correlations of 0.9 for the eye region). This finding raises the question whether face-preference is stable from infancy to adulthood, and if not, which events can modulate this trait. Two studies have examined if infants reliably differ in gaze behavior towards faces. In the first study, toddlers (6–12 months) performed 3 free-viewing tasks that varied in the stimuli type, but always contained a face. They revealed that toddlers that exhibit higher face-preference in one task, also tend to look at faces or to the eye region in the other tasks^59^. In other words, this study showed a high internal consistency of face-preference in toddlers. Another study examined toddlers’ (7 and 24 months) preference looking at faces while distracted by a non-face visual stimulus^60^. Infants’ tendency to fixate on faces at 7 months was related to reduced callous unemotional traits (e.g. lack of empathy) at 48 months of ages. However, the percent of fixation time on faces, while being distracted by a non-face object, was unstable across time. These findings are somewhat indecisive regarding the stability of face preference early in life, but it provides an example for how face-preference is linked to later social behaviors and emotional constructs, such as callous unemotional traits.

Two experiments with two different tasks indicated that individuals vary in the amount of face preference and that this variability is stable across time (hours to weeks). Further research is needed to test the degree to which stability of face-preference extends beyond static images to more naturalistic settings. One recent study found that the position of the first fixation on a face was similar regardless if participants observed faces on a computer screen or if they observed faces when walking down the university corridors^61^. Nevertheless, it is unknown whether individuals’ preference to gaze at faces in general is stable in more ecological tasks, and whether it is related to the face-preference observed when confronted with static images on computer screens.

## Conclusion

Vision is not a passive process, but rather an active process allowing for better sampling of the most interesting aspects in the visual scene. Importantly, people differ in the way they explore the world around them. This study revealed reliable individual differences in face-preference as a proof-of-concept of a novel type of perceptual trait, which determines individuals’ predilection for fixating on specific social content. We report on a stable consistency in face-preference among non-clinical individuals in two tasks (free view and memory task) and across two time intervals (an hour and weeks).

Individual differences in visual exploration and predilection for faces bare immense importance as they reflect differences in top-down control on gaze behavior, which may be related to more general top-down control affecting other cognitive processes, such as attention bias. Critically, differences in gaze behavior not only reflect individual differences in cognitive control^4,62^, they determine the visual input that gets to our brain, and therefore are expected to modulate who we are.

It is important to note that we don’t propose that individual differences are the only factor for selection of gaze positions. Prior research has clearly demonstrated that other factors (the task and the stimuli) also play a crucial role in determining gaze position. Notably, however, individual differences between observers has been understudied. We propose that models for gaze selection would benefit by including the individual preferences as an additional factor. Including face-preference in future models will help to identify effects related to other features, by reducing interindividual variance.

## Methods

### Experiment 1

**Figure 4:**
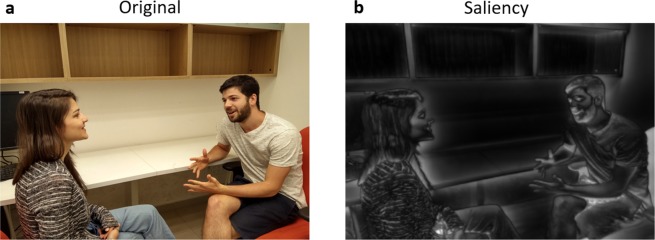
Example picture from experiment 2 and its decomposition to a saliency heatmap. (**a**) Original image. (**b**) Saliency map.

In the first experiment we used images from the image collection published in Xu^29^. Each image in this collection was published together with several heatmaps containing information regarding the semantic aspects of the objects inside. In the current experiment we used the heatmaps that capture information on the locations of faces, in which the center of each face has a maximum value (255) and gradually decreases to zero in a Gaussian manner, when moving away from the center. In our analysis we used a threshold of 64 (as used in Wang 2015) to indicate whether a fixation was directed on a face. Similar threshold and heatmaps were used in this study for saliency^25^. In addition, we created a saliency map, which is a linear combination of the low level features heatmap (orientation, color and intensity) using pySaliencyMap^25,63^ (Fig. 4b; Illustration for saliency map, this image was not included in our experiment).

#### Personality measures

Study participants were assessed for personality using self-reports of the 50-item International Personality Item Pool questionnaire^50^ (IPIP), which measures the personality domains of the Big-Five factor structure: Agreeableness, Conscientiousness, Extraversion, Neuroticism and Openness-to-Experience. Each domain included 10-items rated on a 1–5 scale.

#### Statistical analysis

*Internal consistency – correlations within sessions:* To test whether preference-values are stable within a session we first performed a Pearson correlation across individuals between the values calculated on odd and even trials (split-half reliability). Internal consistency is often used as a measure of the test’s instrumental stability, but it is also a necessary (but not sufficient) factor for the argument that gaze preferences exhibit trait-like patterns. Note however, that these consistencies can be explained by the current state of the observer. Therefore, we also checked the reliability between sessions which are more directly related to traits, rather than states.

*Test-retest reliability – correlations between sessions:* To test whether preferences are stable across time we performed a Pearson correlation across individuals’ preference values in the two testing sessions. High reliability of a preference value suggests that this preference does not meaningfully change from moment to moment and therefore more likely to reflect a trait of the observer.

*Permutation analysis – further examining of the internal consistency and reliability:* Another explanation for face-preference could be the case that observers differ in their preferences to certain facial cues (e.g. eye color) and not to the faces in general. For example, one observer might “prefer” to look at faces with blue eyes and another one might prefer brown eyes. If the faces in our data are not balanced correctly (e.g. all faces with the same eye color) then the variability in face preference could be explained by variability in other facial features.

To examine this option, we performed a permutation analysis in which the data set is divided to 1000 different divisions into two groups, each containing half of the images appeared in each session (20 in experiment 1 and 40 in experiment 2). Then we calculated face-preference for each data-set of images separately in each session. Next, we performed 3 types of correlation analysis: (1) Correlation between face-preference using one data-set and face-preference in the other data-set, within session 1. (2) The same procedure in session 2. (3) Correlation between face-preference in the first data-set in session 1, and face preference in the second data set in session 2. As mentioned, this procedure was performed 1000 times, for each one of the divisions in the permutation procedure.

The results are briefly outlined in the text and expanded in the Supplementary Materials.

*Relations between low level features preference and face-preference:* To control for the influence of saliency-preference on face-preference, we performed partial correlations to check whether the variability in one preference value could be explained by the result of another preference value. Moreover, we performed a paired t-test for each preference value (face and low level saliency) to examine if the values changed in a consistent manner between sessions.

*Correlation with personality measures:* A Pearson correlation was performed between subjects’ face-preference or saliency-preference, averaged across both sessions, and each of the five domains in the Big-Five model.

*Accounting for the effects of recording deviation:* Face-preference values may be attenuated by “recording deviation” of the eye tracker - the distance between where the observer is actually fixating and the predicted fixation position. Observers with relatively large deviations may fixate on a face while the eye tracker would not necessarily report the same result. To measure the recording deviation, we calculated the average distance between the fixation point located in the middle of the screen and the location of the fixation, just before the image was displayed.

To ensure that face-preference differences could not be explained by differences in recording deviation, we performed two analyses in each experiment: (1) We checked the stability of the deviation using subject-wise Pearson correlation across the two sessions. (2) We performed partial Pearson correlations between face-preference in both sessions when controlling for recording deviation. Recording variation did not deviate reliably across sessions and could not explain preference measures, thus results are reported in the Supplementary Materials.

#### Participants

Thirty undergraduate native Hebrew speaking students (Age: M = 25.8, SD = 1.56, all males) took part in this experiment (sample size was based upon previous reports examining the consistency of gaze behavior across time^7^). The experiment was approved by the local ethics committee of the Hebrew University of Jerusalem and conducted in accordance with the Declaration of Helsinki. All participants signed a written informed consent. Two participants were excluded: one for not completing all trials and a second due to a misunderstanding of the task (pressed spacebar to skip trials immediately after the display appeared).

Participants were part of an ongoing study examining correlations between eye movement patterns and stress reactivity. Participants in the current study acted as the control group (data for the stress group is not reported here as is not pertinent for the current hypotheses).

To compute the number of participants, we used ‘pwr’ package in R^64^. In the first experiment we had no prior information on what correlation coefficients to expect. Therefore, we assumed r = 0.5 and power of 0.8 which yield participant number of 30.

#### Procedure

Each participant completed two free viewing sessions taken around an hour apart. We instructed our participant to freely view the images, without any additional instructions or explanations about the rational of this study. In each session participants viewed 40 images containing faces (as defined in the published image collection: Back, profile and frontal faces from humans and animals) with diverse facial features. The same images appeared in session 1 and session 2, each displayed for 3 seconds. Before presenting an image, participants performed a one point drift correction to control for small drifts in the system. Drift correction followed by a fixation point that appeared in the center of the screen until the participant fixated on it for 500 ms. At the break between sessions participants preformed a modified TSST “no audience” control procedure^65,66^. This procedure included (1) reading a magazine article and later reading it aloud, (2) simple counting task, also aloud. Both tasks were performed in an empty room.

Before arriving to the lab, all participants answered a battery of questionnaires including a Big-Five questionnaire. For thorough details of the full procedure see the Supplementary Materials.

#### Eye tracking

Gaze position was tracked using SMI 250RED (SansoMotoric Instruments Inc, Teltow, Germany), installed on DELL laptop. Participants were positioned approximately 60 cm from the monitor. Each participant performed a calibration and validation sessions of five points (implemented by the experimental software provided by SMI) at the beginning of each session. All the data analyzed here was obtained from recordings with an average absolute global validation error of less than 1 degree of visual angle. Recording sample rate was 250hz. The effect of calibration accuracy and dependent variables was assessed and fully described in the Supplementary Materials. Analysis was based on fixations and saccades parceled from the data by the software provided by SMI (BeGaze, SansoMotoric Instruments Inc). Fixations were detected using peak velocity threshold of 40°/s and minimum duration of 50 ms (default SMI implementation). The monitor resolution was 800 × 600 and the stimuli covered the whole screen capturing approximately 24 × 18° of the visual field (at the approximate eye-screen distance of 60 cm).

### Experiment 2

Personality measures and statistical analysis are the same as in experiment 1.

#### Preprocessing the data

In experiment 2 we used a different set of images that contain faces. We defined the faces in the images as Areas Of Interest by drawing circles or rectangles around the faces and heads (AOIs). Using SMI BeGaze program we calculated face-preference as the percent of fixation time on the faces AOIs.

As in experiment 1, we created a saliency map (Fig. 4b) using pySaliencyMap^63^. In experiment 1 we used the feature map existed in the database^29^. In the current experiment we used pySaliencyMap to create feature maps for intensity, color and orientation and calculated the feature-preference values as in experiment 1: the percentage of time gaze was directed on above threshold feature values according to the appropriate heatmap of the image (see methods experiment 1).

#### Participants

Twenty-five undergraduate students (Age: M = 24.22, SD = 2.39, 9 males) took part in this experiment. One participant was excluded because he recognized people that appeared in the images. The experiment was approved by the local ethics committee of the Hebrew University of Jerusalem and conducted in accordance with the Declaration of Helsinki. All participants signed a written informed consent.

In the first experiment we reveal a correlation coefficient of 0.58, therefore in the second experiment we assumed r = 0.58 and in order to have 80% probability to find this effect we needed 20 participants.

#### Procedure

Participants were instructed to memorize 80 images in two separate sessions, performed between 5–30 weeks apart (lag duration was dependent on the participants’ response time to our emails soliciting them to return for a retest). All images included at least two faces that were part of a conflictual situation. The faces varied in their visual characteristics. We selected images of conflictual scenes in order to examine the relation between scanning patterns of conflictual situations and political attitudes (attitudes results will be described in different manuscript). Each image was presented for three seconds and between each image a fixation point appeared at the center of the screen. Before continuing to the next image participants had to look at the fixation point for 500 ms. Following the memorization stage 10 new and 10 old images appeared in a random order and participants were instructed to click on two different keys if they remembered or did not remember the image. After completing the task, participants filled the Big-Five questionnaire and two additional questionnaires that will be discussed in another manuscript with respect to different gaze characteristics (political attitude questionnaire and Interpersonal Reactivity Index^45^).

#### Eye tracking

Gaze position was tracked using SMI 250RED (SansoMotoric Instruments Inc, Teltow, Germany), installed on DELL laptop. Participants were positioned approximately 60 cm from the monitor. Each participant performed a calibration and validation sessions of five points (implemented by the experimental software provided by SMI) at the beginning of each session. All the data analyzed here was obtained from recordings with an average absolute global validation error of less than 1 degree of visual angle. Recording sample rate was 250 hz for 15 participants and 60 hz for 10 participants. Analysis was based on fixations and saccades parceled from the data by the software provided by SMI (BeGaze, SansoMotoric Instruments Inc). Fixations were detected using max dispersion threshold of 2° and minimum duration of 80 ms (SMI implementation). The monitor resolution was 1920 × 1080 and the stimuli covered the whole screen capturing approximately 32 × 18° of the visual field.

## Supplementary information

A novel perceptual trait gaze predilection for faces during visual exploration - supplementary materials
