# Supplementary material for: A novel perceptual trait: gaze predilection for faces during visual exploration
Source: Sci Rep. 2019 Jul 24;9:10714. doi: 10.1038/s41598-019-47110-x (PMC6656722; doi:10.1038/s41598-019-47110-x)
Supplement: Supplementary file 1 — A novel perceptual trait gaze predilection for faces during visual exploration - supplementary materials [file 41598_2019_47110_MOESM1_ESM.docx]

**A novel perceptual trait: gaze predilection for faces during visual exploration**

# Nitzan Guy^1^, Hagar Azulay^1^, Rasha Kardosh^1^, Yarden Weiss^1^, Ran R. Hassin^1^, Salomon Israel^1^, Yoni Pertzov^1^

^1^ The Hebrew University of Jerusalem

**Supplementary:**

**Full procedure of experiment 1**

The pool of participants was based on an ongoing study examining the role of stress reactivity in modulating social behavior and cognition. All participants answered a battery of questionnaires: Beck Depression Inventory^1^ (BDI), Social Phobia Inventory^2^ (SPIN), Social Value Orientation^3^ (SVO), Social Dominance Orientation^4^ (SDO), Autism Quotient^5^ (AQ), Interpersonal Reactivity Index^6^ (IRI) and the Big-Five personality scale. Study participants were randomly assigned to one of two groups: stress group and control group. The stress group underwent the Trier Social Stress Test (TSST), as carried out in previous studies^7^. In brief, the TSST consists of a free speech and a mental arithmetic task performed in front of a panel of a woman and a man with a camera focused on the participant. The participants included in the current experiment were all part of the *control group* that performed a TSST “no audience” control procedure. This procedure included identical instructions and time points as the TSST, however does not include key elements of unpredictability, uncontrollability, or social evaluation which have been previously shown to elicit a stress-response. Each participant completed a free view task twice, once before the control procedure (“session 1”) and once directly after (“session 2”), around an hour apart. In the free view task participants viewed 40 images containing faces as defined in the image collection (Back, profile and frontal faces from human, animals and cartoons). The same images appeared in session 1 and session 2.

**Descriptive tables experiment 1 – preference values**

| Feature | Session | Mean | Std |
| --- | --- | --- | --- |
| Faces | 1 | 42.81 | 7.25 |
|  | 2 | 41.08 | 7.60 |
| Saliency | 1 | 45.36 | 3.18 |
|  | 2 | 44.58 | 3.33 |
| Intensity | 1 | 56.38 | 3.57 |
|  | 2 | 54.97 | 4.76 |
| Color | 1 | 38.74 | 2.88 |
|  | 2 | 37.71 | 4.06 |
| Orientation | 1 | 71.18 | 3.69 |
|  | 2 | 69.46 | 5.18 |

The following table includes the description of face and saliency preferences, means and standard deviations. We measured saliency according to Itti (1998), which suggest that saliency of each pixel is a combination of its spatial contrast in orientation, color and intensity across several scales^8^. Therefore, we added the preference for each of these measures as well.

Table S1: Means and standard deviations of each preference value.

**Pearson correlations of face-preference and saliency-preference with the Big-Five domains in experiment 1**

| Feature | Mean face-preference | Mean saliency-preference |
| --- | --- | --- |
| Extraversion | r(28) = -0.04,  p = 0.824 | r(28) = 0.14,  p = 0.481 |
| Agreeableness | r(28) = 0.01,  p = 0.943 | r(28) = 0. 08,  p = 0. 697 |
| Conscientiousness | r(28) = -0.47,  p = 0.011 | r(28) = -0.12,  p = 0.533 |
| Neuroticism | r(28) = 0.05,  p = 0.785 | r(28) = -0.30,  p = 0.114 |
| Openness-to-Experience | r(28) = -0.21,  p = 0.291 | r(28) = 0.24,  p = 0.225 |

We examined the relation between face and saliency prefernces with Big-Five personality traits. The correlation between conscienenous and face-preference was significant, the others were not.

Table S2: Correlation coefficients between face and saliency preferences and the Big-Five personality traits.

**Pearson correlation of face-preference and saliency-preference with other questionnaires collected in experiment 1**

We examined the relation between face and saliency prefernces with the scores of other questionnaires collected before experiment 1. All the questionnaires, besides SVO, measure the scores on a continous scale. For these questionnaires we performed Pearson correlations between each score and preference values (face and saliency). The results are shown in Figure S3. The SVO questionnaire divides participants to 4 groups according to their social value orientation. In our experiment participants were labeled to only two groups, 24 to one group (prosocial) and 4 to the other (individualistic) ^3^. We performed t-test to compare between groups’ face-preference as well as saliency-preference. The two groups did not significantly differ in their face-preference (t(26) = -1.33, p = 0.19) as well as saliency-preference (t(26) = 0.49, p = 0.63).

Table S3: Correlation coefficients between face and saliency preferences and additional questionnaires included only in experiment 1.

| Feature | Mean face-preference | Mean saliency-preference |
| --- | --- | --- |
| BDI | r(28) = 0.13,  p = 0.51 | r(28) = 0.02,  p = 0.92 |
| SPIN | r(28) = 0.18,  p = 0.36 | r(28) = -0.02,  p = 0.92 |
| SDO | r(28) = -0.04,  p = 0.84 | r(28) = 0.01,  p = 0.96 |
| AQ | r(28) = -0.09,  p = 0.65 | r(28) = -0.19,  p = 0.33 |
| IRI | r(28) = 0.24,  p = 0.22 | r(28) = 0.04,  p = 0.84 |

**Descriptive tables experiment 2 – preference values**

The following table includes the descriptive statistics of face and saliency preferences, means and standard deviations. As in experiment 1 we also included here the preferences of orientation, color and intensity.

Table S4: Means and standard deviations of each preference value.

| Feature | Session | Mean | std |
| --- | --- | --- | --- |
| Faces | 1 | 60.9 | 8.86 |
|  | 2 | 58.73 | 8.52 |
| Saliency | 1 | 60.15 | 3.3 |
|  | 2 | 63.71 | 3.96 |
| Intensity | 1 | 37.15 | 2.47 |
|  | 2 | 40.88 | 3.67 |
| Color | 1 | 30.64 | 3.23 |
|  | 2 | 35.66 | 2.95 |
| Orientation | 1 | 37.55 | 2.54 |
|  | 2 | 40.98 | 4.21 |

**Pearson correlations of face-preference and saliency-preference with the Big-Five domains in experiment 2**

We examined the relation between face and saliency prefernces with Big-Five personality traits. In this experiment participants filled the Big-Five questionnaire twice, once in the first session and the second time between sessions. We used the scores from the second time in our analysis.

| Feature | Mean face-preference | Mean saliency-preference |
| --- | --- | --- |
| Extraversion | r(25) = 0.024,  p = 0.909 | r(25) = 0.039,  p = 0.853 |
| Agreeableness | r(25) = 0.175,  p = 0.403 | r(25) = 0.111,  p = 0.597 |
| Conscientiousness | r(25) = 0.163,  p = 0.436 | r(25) = 0.19,  p = 0.363 |
| Neuroticism | r(25) = 0.296,  p = 0.151 | r(25) = 0.358,  p = 0.079 |
| Openness-to-Experience | r(25) = -0.292,  p = 0.157 | r(25) = -0.253,  p = 0.222 |

Table S5: Correlation coefficients between face and saliency preferences and the Big-Five personality traits.

**Relation between recording deviation and face-preference**

Face-preference is a measure that may be influenced by the “recording deviation” of the eye tracker, defined as the distance between where the observer is fixating and the fixation position that the eye tracker predicts. For example, if tracking an observer’s gaze position has a consistent and large recording deviation, when he\she fixates on a face the eye tracker may report that the fixation is outside of the face – due to the recording deviation. To measure the recording deviation in each trial, we calculated the distance between the fixation point located in the middle of the screen, just before each image appears, and the location of the fixation that the eye tracker provides.

To that end, to ensure that variability in face-preference between individuals is not a result of the variability in recording deviation, we performed two analyses in each experiment:

1. We examined the stability of the deviation across two distinct data sets using the same procedure used for face-preference: a Pearson correlation across individuals’ values.
2. We performed partial correlation between face-preference in both sessions when controlling for the recording deviation in the first session.

**Experiment 1:**

1. The stability of individual differences in recording deviation was assessed by comparing averaged recording deviation in the first and second sessions. The insignificant correlation (r(28) = 0.26, p = 0.177) across individuals suggests that recording deviation is not stable across sessions and therefore unlikely to explain the reliable individual differences in face preference, which are stable across sessions.
2. The correlation between face-preferences in both sessions was still strong and significant, even when controlling for recording deviation (r(25) = 0.601,p = 0.001).

**Experiment 2:**

1. The stability of individual differences in recording deviation was assessed by comparing the averaged recording deviation in the first and second sessions. The insignificant correlation (r(25) = 0.005, p = 0.983) across individuals suggests that recording deviation is not stable across sessions and therefore unlikely to explain the individual differences in face preference, which are stable across sessions.
2. The correlation between face-preferences in both sessions was still strong and significant when controlling for recording deviation (r(22) = 0.548, p = 0.006).

**Permutation analysis to further examine stability of face-preference**

Another potential explanation for individuals’ distinct face-preference could be that certain features of faces, such as blond hair or emotion expression, influence different individuals to different extent. For example, if different individuals exhibit distinct preference to fixate on faces with blond hair, we will measure distinct face preference if all the faces in the data sets have bold hair. In this case, the individual differences would not reflect a general face-preference, but rather a blond hair preference. This concern is valid if the two data sets that are compares consist of similarly unbalanced set of facial features.

To examine this option, without assessing each feature individually (as it is practically impossible to examine all possible face features), we run a permutation analysis. We performed 1000 different divisions of the stimuli into two data-sets, each containing different half of the images that appeared in each session (20 in experiment 1 and 40 in experiment 2). Then we calculated face-preference for each pair of data-sets, separately in each session. Next, we performed 3 types of correlation analysis: 1) Correlation between face-preference in one data-set and face-preference in the other data-set, within session 1. 2) The same procedure in session 2. 3) Correlation between face-preference in the first data-set in session 1, and face preference in the second data set in session 2. This procedure was performed 1000 times, for each one of the divisions in the permutation procedure.


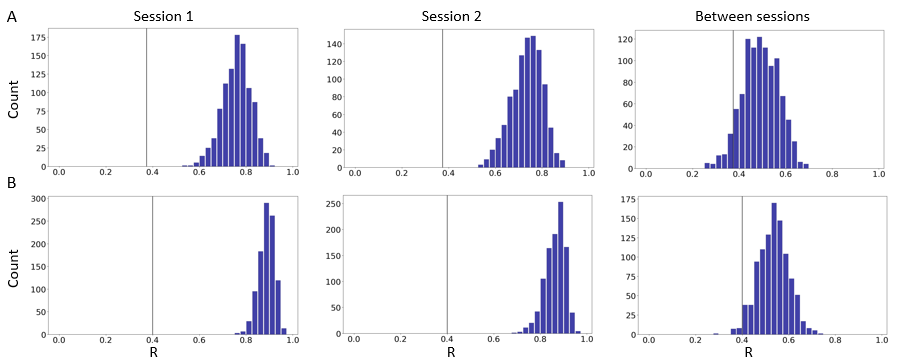
The results showed that internal consistency (correlations within a session) was high, and significantly higher than zero, in all 1000 permutations (experiment 1: mean-r = 0.75, std-r = 0.064; experiment 2: mean-r = 0.88, std-r = 0.04). When we compared between sessions the correlations were also strong (experiment 1: mean-r = 0.49, std-r = 0.077, 93% of permutations were significantly above zero; experiment 2: mean-r = 0.53, std-r = 0.064, 98% of permutations were significantly above zero). Importantly, in experiment 1 we compared two data-sets of 20 images, while in experiment 2 we compared two data-sets of 40 images. This might explain why the correlations in experiment 2 are stronger than in experiment 1. Altogether, we observed high stability between all combinations of data-sets. Therefore, it seems unlikely that individual variability in attraction toward specific facial feature can explain our results.

Figure S6: Permutation analysis for experiment 1 (A) and experiment 2 (B). The permutation analysis was performed 1000 times for sessions 1, session 2 and between both sessions.


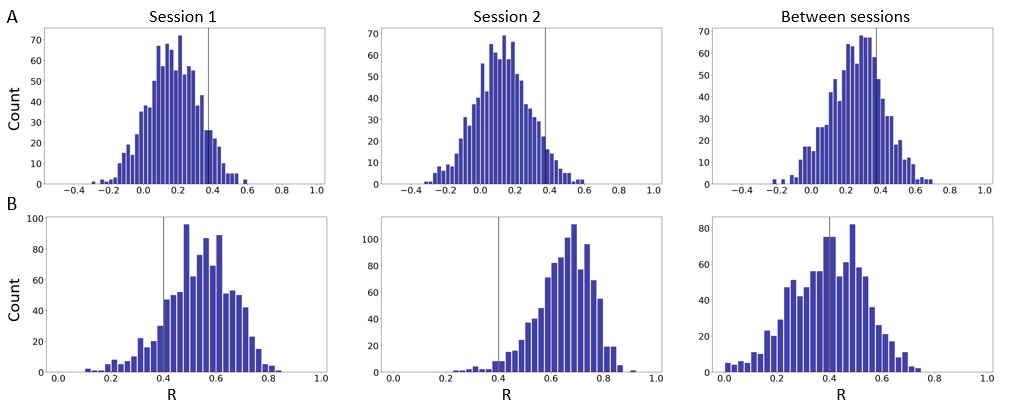
To complete the picture, we performed the same permutation analysis for saliency-preference. Correlations within a session (internal consistency) was high, and significantly higher than zero in most of the permutations of the stimuli in experiment 2 (mean-r = 0.59, std-r = 0.13). However, in experiment 1, we reveal that most of the correlation were not significantly larger than zero (mean-r = 0.15, std-r = 0.15). The difference between the experiment can be a result of the difference set of images in each experiment. The images in experiment 1 were obtained from a set of images which deliberately contains images with differences saliency values. In experiment 2, the images where chosen based on their conflictual situation. When comparison was performed across sessions the correlations were similar to the internal consistency. In experiment 1, most of the correlations were not significantly above zero (mean-r = 0.26, std-r = 0.15) and in experiment 2 half of the correlations were significantly above zero (mean-r = 0.39, std-r = 0.14). Overall, the results suggest that face-preference is more stable than saliency-preference across distinct set of images.

Figure S7: Permutation analysis for experiment 1 (A) and experiment 2 (B). The permutation analysis was performed 1000 times for sessions 1, session 2 and between both sessions.

**Stability of fixation duration, saccade amplitude, saccade velocity and saccadic rate**

We analyzed the stability of basic features of eye movements across individuals, in both experiments. The measures we considered are the mean fixation duration, mean saccade amplitude, mean saccade velocity and mean saccadic rate (saccades per trial).

Similar to Henderson and Luke (2014) we found high internal consistency and reliability in fixation duration and saccade amplitude^9^ (see Table S9 and Table S11 for descriptive). In addition, we found strong stability of saccadic rate (saccades per second) in both experiments. To examine whether the saccadic rate reliability is not dependent on the two known measures (mean saccade amplitude and mean fixation duration), we performed partial correlation and controlled for the average fixation duration and average saccade amplitude. In experiment 1 we revealed strong correlations that we significantly larger than zero when controlling each of these measures (controlling for fixation duration: r = 0.955, p < 0.001; average saccade amplitude: r = 0.957, p < 0.001). However, in experiment 2 the partial correlation was below significance threshold when controlling for the average fixation duration (r = 0.377, p = 0.108) and still significant when controlling for average saccade amplitude (r = 0.639, p = 0.001).

To conclude, as in previous studies, we found that average saccade amplitude and average fixation durations are stable across time. We also suggest saccadic rate as another stable basic feature of eye movements which might be related to fixation durations.


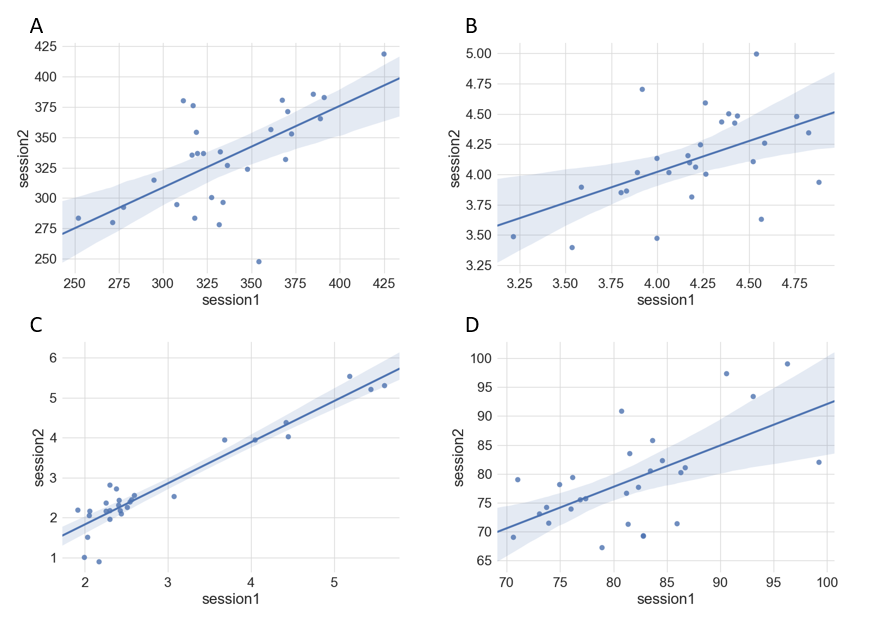
**Experiment 1 – Pearson correlations between session 1 and session 2 in basic eye movement**

Figure S8: Scatter plots, regression line of the relation between session 1 and session 2 in basic eye movement characteristics, each dot represent an individual: (A) average fixation duration. (B) average saccade amplitude. (C) number of saccades per second (D) average saccade velocity

Table S9: Internal consistency and reliability values in experiment 1 for each basic feature of eye movements: fixation duration, saccade amplitude, saccadic rate and average velocity.

| Basic eye movement measure | Internal consistency  session 1 | Internal consistency  session 2 | Reliability between sessions |
| --- | --- | --- | --- |
| Fixation duration | r = 0.51, p = 0.005 | r = 0.51, p = 0.005 | r = 0.63, p < 0.001 |
| Saccade amplitude | r = 0.58, p = 0.001 | r = 0.60, p < 0.001 | r = 0.52, p = 0.004 |
| Saccadic rate | r = 0.98, p < 0.001 | r = 0.98, p < 0.001 | r = 0.95, p < 0.001 |
| Average velocity | r = 0.66, p < 0.001 | r = 0.68, p < 0.001 | r = 0.61, p < 0.001 |


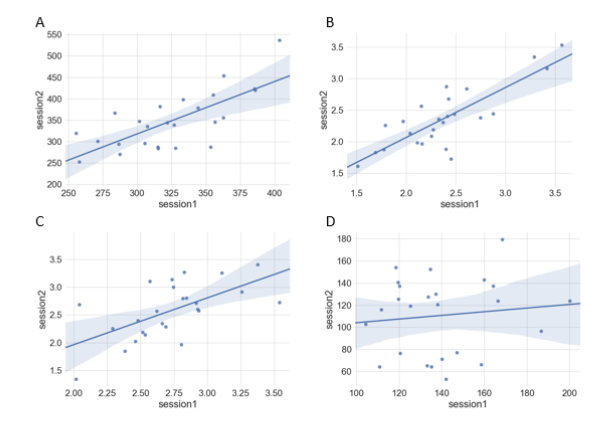
**Experiment 2 – Pearson correlations between session 1 and session 2 in basic eye movement characteristics:**

Figure S10: Scatter plots and regression line of the relation between session 1 and session 2 in basic eye movement characteristics: (A) average fixation duration. (B) average saccade amplitude. (C) saccades per second (D) avg saccade velocity

Table S11: Internal consistency and reliability values in experiment 2 for each basic feature of eye movements: fixation duration, saccade amplitude, saccadic rate and average velocity.

| Basic eye movement measure | Internal consistency  session 1 | Internal consistency  session 2 | Reliability between sessions |
| --- | --- | --- | --- |
| Fixation duration | r = 0.90, p < 0.001 | r = 0.96, p < 0.001 | r = 0.73, p < 0.001 |
| Saccade amplitude | r = 0.92, p < 0.001 | r = 0.87, p < 0.001 | r = 0.82, p < 0.001 |
| Saccadic rate | r = 0.91, p < 0.001 | r = 0.96, p < 0.001 | r = 0.62, p = 0.001 |
| Average velocity | r = 0.94, p < 0.001 | r = 0.97, p < 0.001 | r = 0.12, p = 0.584 |

**Entry time to face region**

During the review process we found out about a relevant pre-print published in bio-archives^10^. This study examined gaze behavior during initial stages of scene viewing and its relation to face recognition. More specifically, they measured the reliability of the percent of first fixations on face regions and found this measure to predict face recognition performance in another task. To correspond with this article, we also perform similar analysis. However, we do not perform the same analysis, because the percent of first fixations is widely affected by whether you include short fixations which occur near the fixation point presented before image onset. Therefore, we measured the average time it took gaze to fall to the face regions.

To check the reliability of this measure between sessions, we performed two Pearson correlations, one for each experiment. The correlation in experiment 1 was significantly larger than zero (r = 0.38, p = 0.043) and marginally significant in experiment 2 (r = 0.36, p = 0.076). Our results suggest that face-preference is more stable than the mean entry time to face regions.


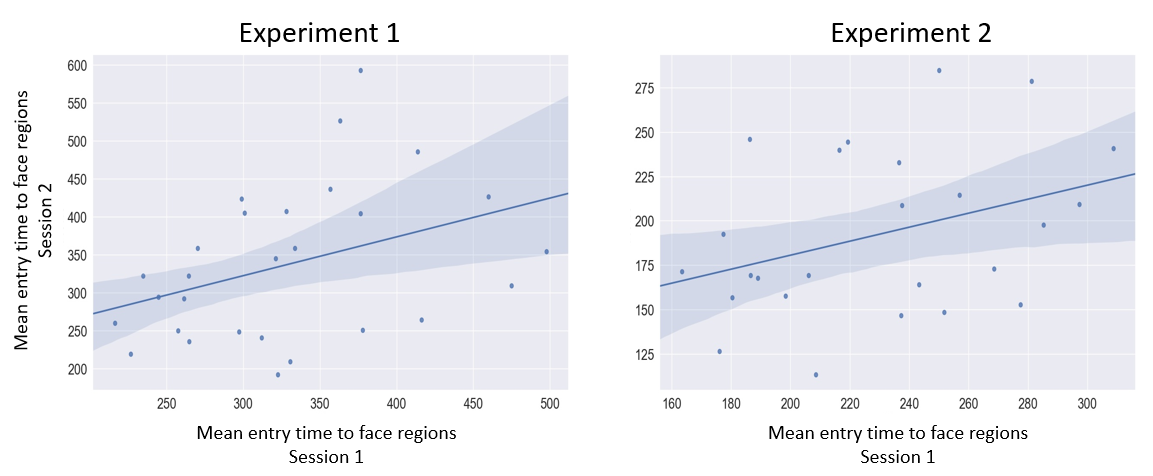


Figure S12: Scatter plots and regression lines between the mean entry time to face regions in session 1 and session 2. Experiment 1 (left) and experiment 2 (right)

**References:**

1. Beck, A. T., Steer, R. A. & Brown, G. K. Beck depression inventory-II. *San Antonio* **78**, 490–498 (1996).

2. Connor, K. M. *et al.* Psychometric properties of the Social Phobia Inventory (SPIN): New self-rating scale. *The British Journal of Psychiatry* **176**, 379–386 (2000).

3. Murphy, R. O., Ackermann, K. A. & Handgraaf, M. Measuring Social Value Orientation. *SSRN Electronic Journal* (2011). doi:10.2139/ssrn.1804189

4. Pratto, F., Sidanius, J., Stallworth, L. M. & Malle, B. F. Social dominance orientation: A personality variable predicting social and political attitudes. *Journal of Personality and Social Psychology* **67**, 741–763 (1994).

5. Baron-Cohen, S., Wheelwright, S., Skinner, R., Martin, J. & Clubley, E. The autism-spectrum quotient (AQ): Evidence from asperger syndrome/high-functioning autism, malesand females, scientists and mathematicians. *Journal of autism and developmental disorders* **31**, 5–17 (2001).

6. Davis, M. H. A multidimensional approach to individual differences in empathy. (1980).

7. Kirschbaum, C., Pirke, K. M. & Hellhammer, D. H. The ’Trier Social Stress Test’--a tool for investigating psychobiological stress responses in a laboratory setting. *Neuropsychobiology* **28**, 76–81 (1993).

8. Itti, L., Koch, C. & Niebur, E. A model of saliency-based visual attention for rapid scene analysis. *IEEE Transactions on pattern analysis and machine intelligence* **20**, 1254–1259 (1998).

9. Henderson, J. M. & Luke, S. G. Stable individual differences in saccadic eye movements during reading, pseudoreading, scene viewing, and scene search. *Journal of Experimental Psychology: Human Perception and Performance* **40**, 1390–1400 (2014).

10. de Haas, B., Iakovidis, A. L., Schwarzkopf, D. S. & Gegenfurtner, K. R. Individual differences in visual salience vary along semantic dimensions. *bioRxiv* (2018). doi:10.1101/444257
